# Supplementary material for: The antenatal psychological experiences of women during two phases of the COVID-19 pandemic: A recurrent, cross-sectional, thematic analysis
Source: PLoS One. 2023 Jun 8;18(6):e0285270. doi: 10.1371/journal.pone.0285270 (PMC10249846; doi:10.1371/journal.pone.0285270)
Supplement: Attachment — Submitted filename: PRaM Prenatal Psych - Response to Reviewers.docx [file pone.0285270.s001.docx]

| **Point-by-Point Response**  ***PLoS One* – PONE-D-23-01119** | |
| --- | --- |
| **Editor’s Comments** | |
| Please address the reviewer's comments as best as possible, as I will strongly consider them in my decision. | We believe we have made all the necessary changes upon the advise of the Reviewer and yourself as Editor. |
| I noted in my review that the paper only interviewed women in the third trimester and wanted to know why a broader sample across pregnancy stages wasn't considered. Pregnancy experiences vary between pregnancy stages and also between first and second time mothers-the latter I felt needs to be addressed in the paper. | Thankyou for raising this concern. We have added an explanation to the paper, that this allowed for women to have experienced some level of maternity care prior to the pandemic restrictions being emplaced. |
| The chosen sample of respondents must also be carefully interpreted, given the selective sampling frame which influences the respondent's insights. An added perspective could be identifying how ethnicity shaped the respondent's experience, which would be insightful to potential readers. | We believe ethnicity has an extremely important role in studies such as this – particularly during the pandemic – we do not believe there are enough participants or variation amongst them to make a meaningful argument about ethnicity, hence it has not been included in our analyses. |
| Table 2 is useful to get a sense of broad UK restrictions; however, it's left to the reader to interpret the degree of disruption subjectively these may have caused. Considering our international readership, please contextualise the health system and social disruption pertinent to the study population. | On reflection we do not consider Table 2 to be a useful addition to the paper and have therefore removed it. |
| In conclusion, I would also encourage the authors to reflect that the impact of the pandemic lockdown was seen differently in 2020 compared to later years when vaccination was available as an intervention. Given the evolution of the pandemic over three years(2020-2023) and our understanding, you may like to provide some lines reflecting on these changes and how the study results are retrospectively viewed. | Thankyou for this helpful suggestion. We have added an entire section to the Strengths, Limitations, and Future Directions section. |
| **Reviewer 1’s Comments** | |
| Thank you for the opportunity to review this interesting paper. | We would like to thank the reviewer for their time and for their positive appraisal of our manuscript. |
| Why would you compare pregnant women to nulliparous adults? Would you not compare them to non-pregnant people? | We thank the reviewer for posing this question. We have gone back to the literature from which the point has been made and actually have deleted the comparator statement, to be a more accurate reflection of the published work. |
| 1.2 I think you need a bit more of the way the disease was spreading prior to the pandemic declaration, currently the text goes form the first case to pandemic. At the time the Public Health Emergency of International Concern was a big deal.  The WHO in China first noted reports of viral pneumonia in Wuhan on 31st December 2019. I think you should identify the rapid spread of the disease and that by 31st Jan 2020 the WHO declared a Public Health Emergency of International Concern (WHO Situation report no.11), by the time the pandemic was declared on 11th March (WHO satiation report no. 51) there were more than 118,000 cases and almost 4300 deaths in 23 countries. | Thankyou for this suggestion, we have now indeed included more information in this section |
| 1.3 could “Antenatal and 0-5 services…” be rephrased to read more clearly: Antenatal and services for children aged 0-5…… | Thankyou for suggesting this, we have now amended both accounts of this. |
| The phrase “, …was ineffective for building the necessary report to disclose…” probably should state rapport | Thankyou for spotting this and apologies for missing it before submission! |
| Informal support (eg maternal grandmother)- could this not be broadened to grandparents? Might be more inclusive. | We agree with you and have amended accordingly |
| 2. The paragraph about participation exhaustion making recruitment difficult and yet you state previously that recruitment was over-subscribed. This would appear to be contradictory. I am confused regarding the need for the additional recruitment strategy and that some women were recruited in September. | We have made this clearer. |
| Time point 1 was March 2020 and time point 2 was July 2020 (when restrictions lifted). As it was a one off interview what was the issue with attrition? Could you not have gone back to the random generator and recruited other women? How postnatal were the postnatal women? Obviously this is a huge source of potential recall bias, was this strategy really the most effective given the initial interest from 72 women (3 times the number actually interviewed)? | We have made this whole section clearer as to why some postnatal women were included. |
| Table 1. Interesting that only 3 women did not have a degree at all and everyone at T2 had at least one! Overall 25% had Doctorates (6/24) although usually samples often contain those with highest educational attainment, this group would appear particularly so. Interesting when compared to the UKOSS evidence around women who got COVID. | Thankyou for raising this. It is a problem with the nature of on-line recruitment – we have added this to the limitations too |
| Is there a typo in time point 1 Ceinwen ‘s quote? | We have corrected this – thanks for spotting it. |
| Discussion: Many of the quotes and feelings could have been due to the pregnancy per se. Overall the findings are appropriately presented and discussed | We thank the reviewer for their positive appraisal of our results. |
| Strengths and Limitations: The authors acknowledge this study adds to the ‘substantial body of literature around untailored social distancing legislation” and the ‘dire consequences’ for the mental health of pregnant women. But is this different from the catastrophic impact on mental health social distancing had for the general population, and the body of evidence around this? It could be argued the additional time to look after themselves and bond with their unborn child might counterbalance the impact of social isolation. | Thankyou for this suggestion, we have incorporated |
| I still think further explanation is required around attrition given the positive spin around initial over recruitment. You have not discussed the impact of potential bias associated with recruiting in this way or dealing with the attrition. Interestingly the lack of “baby showers” and other social rituals around pregnancy indicate how important these are but that ‘baby showers’ are a relatively recent invention and yet clearly are the ‘norm’ for women in pregnancy now. | We have reiterated the issues here in an expanded section of the limitations. |
| When you conclude that most respondents experienced deterioration in mental health during the pandemic, could this be due to the pregnancy, or be due to the same influences as the general population? | Thankyou for this suggestion. We have provided an expansion of this section to further explain. |
