# Supplementary material for: The antenatal psychological experiences of women during two phases of the COVID-19 pandemic: A recurrent, cross-sectional, thematic analysis
Source: PLoS One. 2023 Jun 8;18(6):e0285270. doi: 10.1371/journal.pone.0285270 (PMC10249846; doi:10.1371/journal.pone.0285270)
Supplement: Attachment — Submitted filename: Example of thematic table.docx [file pone.0285270.s002.docx]

Example of a table showing both T1 and T2 themes in one place for reader comprehension

| thematic structure | | | |
| --- | --- | --- | --- |
| Time period One Mar 2020 (T1) | | Time period Two July 2020 (T2) | |
| Theme | Subtheme | Theme | Subtheme |
| 1. A Mindful Pregnancy | 1.1 Listening to Your Physical and Emotional Needs | 1. Coping with Lockdown Restrictions | 1.1. A slower pace of life |
|  | 1.2. Controlling What You Can and Disengaging from What You Can’t |  | 1.2. Being kind and keeping structure |
| 2. It’s a Grieving Process | 2.1. Lonely and Anxious | 2 Robbed of our Pregnancy | 2.1. Unmet expectations and feeling guilty |
|  | 2.2. Lost Freedoms and Unmet Expectations |  | 2.2 The voice of others feels loud |
|  |  |  | 2.3. Left in the dark |
