# Supplementary material for: The antenatal psychological experiences of women during two phases of the COVID-19 pandemic: A recurrent, cross-sectional, thematic analysis
Source: PLoS One. 2023 Jun 8;18(6):e0285270. doi: 10.1371/journal.pone.0285270 (PMC10249846; doi:10.1371/journal.pone.0285270)
Supplement: Attachment — Submitted filename: PRaM Prenatal Psych - Response to Reviewers v.2.docx [file pone.0285270.s003.docx]

| **Point-by-Point Response**  ***PLoS One* – PONE-D-23-01119** | |
| --- | --- |
| **Editor’s Comments** | |
| I thank the authors for working on the editor and reviewer comments and providing a revised manuscript. I can recommend it for publication barring one additional change I encourage the authors to make in another revised submission which would receive an expedited review. Reading the results section and themes linked to the period provides key insights. While the authors flag the timepoint, it is slightly confusing in the final revised paper to know which theme/subtheme was linked to the particular period, especially for T2 themes number two onwards. Moreover, the results haven't currently provided both time point 1 and 2 themes together, allowing for side-by-side comparison. While the discussion elaborates on themes, visually comparing both themes allows for greater comprehension of the results and enhances linkages in the results section  I suggest the authors add a table providing T1 and T2 themes together in the results section, preferably before the T1 theme's results start. For the author's reference, I have attached an example of such a table. Please consider providing a few additional lines in the results section to start introducing the table and providing readers with information on how the results section is presented viz that the results first provides time period themes one followed by period two themes etc | We thank the Editor for this suggestion. We have made the timepoints clearer in both the abstract and the methods section, and – as requested – have added a table of themes as per the timepoints into the results section. |
